# Hypertension Caused by Lenvatinib and Everolimus in the Treatment of Metastatic Renal Cell Carcinoma

**DOI:** 10.3390/ijms18081736

**Published:** 2017-08-10

**Authors:** Mathias Alrø Fichtner Bendtsen, Daniela Grimm, Johann Bauer, Markus Wehland, Petra Wise, Nils E. Magnusson, Manfred Infanger, Marcus Krüger

**Affiliations:** 1 Institute of Biomedicine, Pharmacology, Aarhus University, Wilhelm Meyers Allé 4, DK-8000 Aarhus C, Denmark; bendtsen_mat@hotmail.com; 2 Max Planck Institute for Biochemistry, Am Klopferspitz 18, 82152 Martinsried, Germany; jbauer@biochem.mpg.de; 3 Clinic and Policlinic for Plastic, Aesthetic and Hand Surgery, Otto von Guericke University, Leipziger Str. 44, 39120 Magdeburg, Germany; markus.wehland@med.ovgu.de (M.W.); manfred.infanger@med.ovgu.de (M.I.); marcus.krueger@med.ovgu.de (M.K.); 4 Hematology/Oncology, University of Southern California, Children’s Hospital Los Angeles, 4650 Sunset Blvd. MS #57, Los Angeles, CA 90027, USA; pwise@chla.usc.edu; 5 Medical Research Laboratory, Department of Clinical Medicine, Faculty of Health, Aarhus University, Nørrebrogade 44, DK-8000 Aarhus C, Denmark; nm@clin.au.dk

**Keywords:** metastatic renal cell carcinoma, lenvatinib, everolimus, hypertension, multikinase inhibitors, mTOR inhibitor, vascular endothelial growth factor

## Abstract

Multikinase inhibitors (MKI) and mammalian target of rapamycin (mTOR) inhibitors prolong progression-free (PFS) and overall survival (OS) in the treatment of metastatic renal cell carcinoma (mRCC) by reducing angiogenesis and tumor growth. In this regard, the MKI lenvatinib and the mTOR inhibitor everolimus proved effective when applied alone, but more effective when they were administered combined. Recently, both drugs were included in clinical trials, resulting in international clinical guidelines for the treatment of mRCC. In May 2016, lenvatinib was approved by the American Food and Drug Administration (FDA) for the use in combination with everolimus, as treatment of advanced renal cell carcinoma following one prior antiangiogenic therapy. A major problem of treating mRCC with lenvatinib and everolimus is the serious adverse event (AE) of arterial hypertension. During the treatment with everolimus and lenvatinib combined, 42% of the patients developed hypertension, while 10% of the patients treated with everolimus alone and 48% of the of the lenvatinib only treated patients developed hypertension. Lenvatinib carries warnings and precautions for hypertension, cardiac failure, and other adverse events. Therefore, careful monitoring of the patients is necessary.

## 1. Introduction

Angiogenesis is essential for the progression of renal cell carcinoma (RCC) tumor growth and metastasis. The process of neoangiogenesis is critical for tumor growth and progression and is therefore an important target in cancer therapy [1].

In recent years, considerable efforts were made to prevent tumor-induced angiogenesis by developing strategies of dysregulating the relevant signaling pathways. Now inhibitors of these angiogenetic signaling pathways are available and may be used as therapeutic agents in RCC [2,3,4]. Two of the most used representatives are the multikinase inhibitor (MKI) lenvatinib and the mTOR (mammalian target of rapamycin) inhibitor everolimus.

Both types of drugs have been tested extensively in clinical trials, confirming that everolimus alone prolongs progression-free survival (PFS) and that everolimus and lenvatinib combined improve PFS and overall survival (OS) in patients with advanced RCC [5]. Therefore, the American Food and Drug Administration (FDA) approved the combination of lenvatinib and everolimus as treatment for metastatic RCC (mRCC) [6]. Everolimus is also indicated for the treatment of mRCC during disease progression, regardless of prior anti-angiogenic therapy [7,8].

However, both drugs do have adverse effects. The FDA lists various critical adverse events (AE) for the treatment with a combination of everolimus and lenvatinib. They comprise hypertension, nausea and vomiting, lowered appetite, weight loss, gastrointestinal problems such as diarrhea or abdominal pains, dyspnea, cough, stomatitis, proteinuria, hemorrhagic events, joint and muscle pain, fatigue, edema and rash [6]. Hypertension is particularly emphasized.

Hypertension is a common AE of a combined treatment with everolimus and lenvatinib (43%), whereas 10% of the patients treated with everolimus alone and 48% of the patients treated with lenvatinib alone develop hypertension [9]. Therefore, the FDA flags hypertension as an AE of lenvatinib and recommends to control blood pressure prior to treatment with lenvatinib and to withhold lenvatinib when grade 3-hypertension occurs, despite an optimal antihypertensive treatment [9]. Finally, there is a FDA precaution to discontinue lenvatinib in case of a life-threatening hypertension.

**Figure 1:**
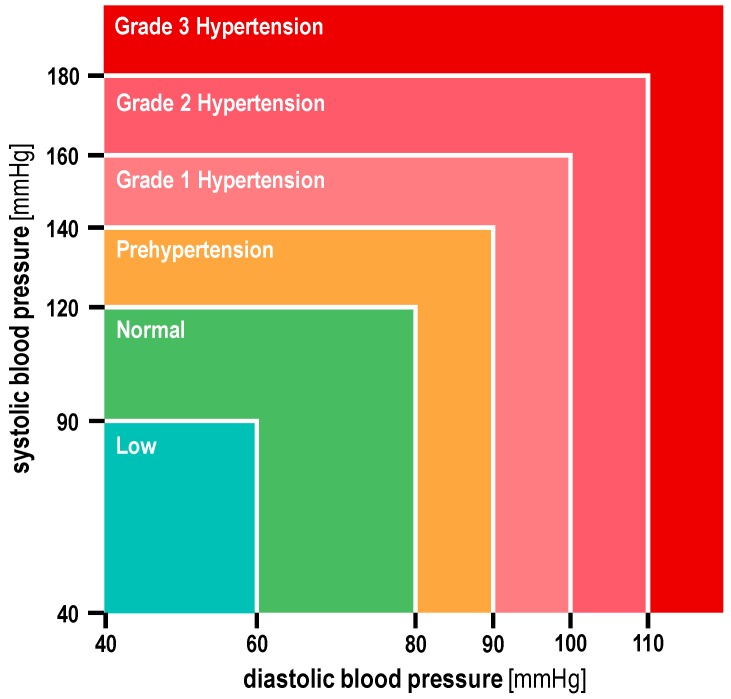
Different grades of arterial hypertension.

Hypertension is subdivided into different grades (Figure 1). Grade 2-hypertension is critical while grade 3-hypertension poses a serious threat to patients. A systolic blood pressure ≥160 mmHg occurred in 29% of patients and 21% of patients had a diastolic blood pressure ≥100 mmHg (grade 2-hypertension) in the group treated with the combination therapy [9]. Grade 3-hypertension occurred in 13% of patients receiving combination therapy, while 2% of patients treated with everolimus alone had grade 3-hypertension.

In this review, we focus on the adverse effect of hypertension, which frequently arises during treatment of mRCC with everolimus or combined everolimus and lenvatinib. In particular, we will try to answer these questions:

1. What is the positive effect of combined treatment with everolimus and lenvatinib for RCC?
2. How can everolimus and lenvatinib cause hypertension during treatment for RCC?
3. What is the negative effect caused by hypertension on patients treated with everolimus and lenvatinib combined?
4. Does the net effect favor the combination of the drugs?
5. Can the adverse effects caused by hypertension induced by everolimus and lenvatinib be modified?
6. What is the best alternative of treatment?

Answering these questions may close our current knowledge gap and help optimize the treatment of patients with advanced RCC.

Literature and information used for this review was retrieved using online databases, such as Pubmed, Scopus and clinicaltrials.gov by using the search terms “multikinase inhibitors”, “antiangiogenesis”, “multikinase inhibitors and hypertension”, “renal cell carcinoma” and others.

## 2. Metastatic Renal Cell Carcinoma

With an incidence of 90–95%, RCC constitutes the majority of kidney cancers. It is defined as a cancer originating from the epithelial cells lining the proximal convoluted tubules in the renal cortex. There is a sporadic form and an inherited form. The sporadic form is typically a solitary tumor that is especially prevalent among older male cigarette smokers [10]. The inherited von Hippel-Lindau (VHL) disease is typically bilateral and affects men and women equally. It is due to a rare autosomal dominant mutation [11] and represents 2–3% of all RCC. The most common subtypes of the hereditary form are von Hippel-Lindau, hereditary papillary RCC, and hereditary leiomyomatosis RCC [12]. In this case, the patients suffer from a germline mutation in the *VHL* gene, located on 3p25, which inactivates the allele. When the wild-type allele is lost, the gene product pVHL is no longer produced. The pVHL protein works as a substrate for the E3 ubiquitin ligase complex that induces the hypoxia-inducible factor for degradation due to polyubiquitination [13]. The loss of the *VHL* gene results in a greater transcription of hypoxia-inducible factor (HIF) genes. Additionally, the VHL tumor suppressor gene inhibits the expression of the chemokine receptor type 4 (CXCR4) by degrading HIF, which promotes transcription of CXCR4. Thereby, the loss of *VHL* results in increased chemotaxis and risk of metastatic spread. This increases the amount of vascular endothelial growth factor (VEGF), platelet-derived growth factor (PDGFα), transforming growth factor (TGFα) and erythropoietin [14].

## 3. Therapy of Renal Cell Carcinoma

As the number of available drugs and related research has grown continuously, treatment options for RCC changed dramatically during the last years. Every treatment of RCC depends on the TNM staging (tumor growth locally (T), spread to retroperitoneal lymph nodes (N), and metastases to other organs (M)). Tumor growth locally is ranked from 0 to 4, where grade 4 is the most severe. The spread to retroperitoneal lymph nodes are ranked from 0 to 2. Metastases to other organs are ranked from 0 to 1 [15]. If the tumor is localized in the kidney and has not spread to lymph nodes or metastasized, surgical resection of the kidney is the treatment of choice [16], because RCCs are refractory to traditional oncological therapy such as chemotherapy and radiation. Only sometimes the RCC tumor is sensitive to immunomodulatory agents such as various chemokines and antibodies [17].

In many cases the RCC develops into the metastatic form mRCC, invading the renal veins followed by systemic spread of metastases to other organs such as the lungs and bones [12,18]. Among the mRCCs the clear cell RCC (ccRCC) is by far the most common subtype. It represents 83–86% of mRCC. mRCCs which are not ccRCC are denoted non-clear-cell RCC for convenience during clinical studies [18]. The VHL-associated RCC has more or less the same pathogenesis as most of the sporadic ccRCC.

If surgical resection is not possible, in most cases mRCC tumors are treated with molecular targeted therapy—especially with inhibitors of VEGF receptors [3,4,19]. There are five isoforms of VEGF as well as three VEGF receptors, which can all be targets of VEGF inhibition [20]. Binding of VEGF to its receptors leads to an autophosphorylation of the receptor tyrosine kinase (RTK) which results in a signal cascade that involves Ras protein, Raf proto-oncogene serine/threonine-protein kinase (RAF-1), mitogen-activated protein kinases (MEK), extracellular signal-regulated kinases (ERK), phosphatidylinositol 3-kinase (PI3K) and phospholipase C (PLC). Activation of the Raf/MEK/ERK cascade results in cellular proliferation, differentiation, angiogenesis, adhesion, cell mobility and prolonged cellular survival. Up-regulation of the Raf/MEK/ERK cascade increases the risk of tumorigenesis and progression [21]. Inhibition of VEGF-dependent signaling cascades reduces tumor vascularization, which inhibits tumor growth and provides tumor shrinkage in experimental models [20,22,23]. Typical inhibitors of VEGF cascades are lenvatinib, sorafenib, sunitinib, pazopanib, axitinib, or cabozantinib. In this review, we focus on lenvatinib [9]. It inhibits the intracellular kinase activity of the vascular endothelial growth factor (VEGF) receptors VEGFR1, VEGFR2, VEGFR3 as well as other RTKs involved in pathogenic neoangiogenesis, tumor growth, and metastasis in RCC.

Further molecular targets of tumor cell growth cascades include the mammalian target of rapamycin (mTOR) pathway. mTOR is a serine/threonine-specific protein kinase, which enhances cell metabolism, growth, and proliferation by generating two protein complexes mTORC1 and mTORC2 that include mTOR itself. The mTORC1 and mTORC2 protein complexes activate protein translation and are inhibited by everolimus, a rapamycin derivative. The inhibition of mTOR retards cell growth and induces inhibition of HIF [24].

### 3.1. Therapeutic Effects of Everolimus and Lenvatinib

**Figure 2:**
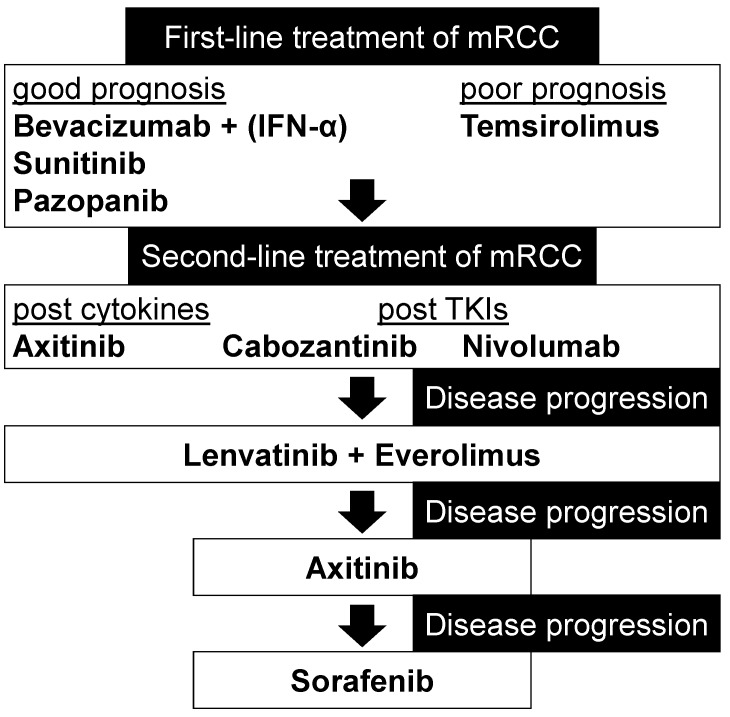
First- and second-line treatment of mRCC, modified from [15,27].

Lenvatinib and everolimus are used as the second-line treatment of mRCC (Figure 2). The antiangiogenic and antitumor activities of lenvatinib alone are insufficient in treating mRCC. However, the activities are enhanced by combination with the mTOR inhibitor everolimus. In mouse xenografts of human RCC, the combination of lenvatinib and everolimus inhibited the human endothelial cell growth, tube formation, VEGF signaling and tumor growth more than each drug alone [9,25,26].

mTOR inhibitors approved for first-line and second-line treatment of mRCC are everolimus and temsirolimus. First-line treatment with mTOR inhibitors is only recommended to patients with a poor prognosis [28,29,30,31,32,33]. In March 2009, the FDA approved everolimus as second-line treatment of advanced RCC, when first-line treatment had failed [6]. Especially, if first-line therapy with a VEGF targeted agent has failed, everolimus is approved for second-line therapy of patients with mRCC (Figure 2). Evidence supports that everolimus improves median PFS by approximately 2 months compared to placebo after failure of first-line therapy, but shows no therapeutic effect compared to placebo as first-line therapy [30].

Both PFS and OS are improved by combining everolimus and lenvatinib compared to a monotherapy with everolimus as second-line treatment. A multicenter phase II randomized controlled trial (RCT) found that median PFS with combined everolimus and lenvatinib was 14.6 months compared to 5.5 months with everolimus as second-line monotherapy (HR: 0.40; 95% CI: 0.24–0.68; *p* = 0.0005) [31]. In a retrospective, blinded review, it was found that in the group receiving the combination therapy of lenvatinib and everolimus the PFS was significantly improved compared to the group receiving everolimus alone [34]. A phase I trial [35] has explored the effect of a therapy of mRCC with a combination of lenvatinib and everolimus as second-line treatment [36]. The maximum tolerated dose (MTD) was 18 mg lenvatinib/5 mg everolimus per day. The rate of partial response was 30% (95% CI: 11.9–54.3%). The median PFS was 330 days (95% CI: 157–446 days). The 6 months PFS rate was 72.1% (95% CI: 48.8–95.4%) and 12 months PFS rate was 49.5% (95% CI: 22.7–76.2%).

Hypertension was among the most frequent AE with an incidence of 40%. A phase II RCT demonstrated a significant improvement of PFS from 5.5 months with everolimus as monotherapy to 14.6 months with combined lenvatinib and everolimus (HR 0.40; CI: 0.24–0.68) in mRCC after failed previous VEGF monotherapy [31]. In this trial, combined lenvatinib and everolimus did not improve PFS compared to lenvatinib as monotherapy for second-line treatment (HR 0.66; CI: 0.39–1.10; *p* = 0.12). No significant difference in OS was demonstrated between the three study groups (combined lenvatinib/everolimus vs. lenvatinib vs. everolimus). Table 1 summarizes the most relevant clinical trials administering lenvatinib and everolimus to patients with RCC.

### 3.2. Adverse Events of Everolimus and Lenvatinib

The treatment with lenvatinib is known to significantly increase the risk of both all-grade hypertension (47%) and high-grade hypertension (17.7%). It is therefore mandatory to monitor BP of patients receiving lenvatinib and to treat with antihypertensive agents or to reduce the dose if necessary [38]. Frequent grade 3 AE associated to lenvatinib treatment of RCC are hypertension (2%), nausea (8%, all grades 62%), diarrhea (2%, all grades 72%), myalgia (2%, all grades 14%), and fatigue (8%, all grades 50%) [9,39,40]. Hypertension occurs in 15–60% of patients when treated with VEGF kinase inhibitors [20]. Everolimus alone can cause hypertension (2% grade 3/4 vs. 10% all grades), fatigue (40% vs. 2%), diarrhea (34% vs. 2%), renal failure (12% vs. 2%), hemorrhagic events (26% vs. 2%), nausea (16% vs. 0%), myalgia (32% vs. 0), and oral inflammation (16% vs. 0%). A combination therapy with both drugs significantly increases the risk for grade 3/4 AEs, while rates for all grade AEs remain mostly unchanged or are only slightly increased in comparison to the single agent therapies. The most frequent AEs are hypertension (42% all grades vs. 13% grade 3/4), fatigue (73% vs. 18%), diarrhea (81% vs. 19%), renal failure (18% vs. 10%), hemorrhagic events (32% vs. 6%), nausea (45% vs. 5%), myalgia (55% vs. 5%), and oral inflammation (44% vs. 5%).

### 3.3. Hypertension During Treatment of Metastatic Renal Cell Carcinoma

Hypertension is a frequent and severe AE, when VEGF inhibitors are applied as an antiangiogenic agent [20,41,42], whereas it is less frequent and less severe with mTOR inhibition. Hypertension as an AE of combined VEGF kinase and mTOR inhibition is mainly due to VEGF kinase inhibition [24,43]. The mechanism of the hypertensive effect of VEGF inhibitors is complex. VEGF enhances the activity of endothelial nitric oxide synthase (eNOS). This effect has been demonstrated by in vitro models with human umbilical vein endothelial cells (HUVEC). Nitric oxide (NO) relaxes baseline smooth muscle vascular tone [20]. VEGF also leads to an increased prostacyclin production and release. Prostacyclin decreases baseline vascular tone—i.e., it decreases vascular resistance and blood pressure [20]. This means that inhibition of the VEGF pathway decreases production and release of NO and of prostacyclin, but increases vascular resistance and blood pressure [20,44].

VEGF inhibition also causes vascular endothelial cell apoptosis in non-tumor tissue and thereby reduces the number of small arteries, arterioles and capillaries. This phenomenon is called rarefaction and is divided into structural and functional rarefaction. Structural rarefaction is the disappearance of microvessels, while functional rarefaction is rendering capillaries non-perfusable [18,20,44]. The decrease in small vasculature causes a rise in blood pressure, which leads to hypertension.

Blocking of VEGFRs inhibits angiogenesis and growth of capillaries and induces hypertension. No evidence has elucidated whether the rarefaction is structural or functional or a combination of both, although the sudden reversal of hypertension in patients taken off VEGF-inhibitors suggests a functional rarefaction [20,45]. Clinical data suggest that VEGF kinase inhibition increases stiffness in the aorta and the main aortic branches, without affecting known hypertensive renovascular effectors such as serum renin, aldosterone, and catecholamine [20]. A proposed mechanism is that NO deficiency enhances vascular medial cell proliferation [2]. A reduced production of NO increases renal sodium retention and thereby arterial blood pressure [2].

### 3.4. Efficacy of Treatment of Metastatic Renal Cell Carcinoma with Multikinase Inhibitors versus the Hypertensive Effect

The clinical problem with a monotherapy of mRCC is that almost all patients develop resistance to it, leading to a progression of tumor growth and metastases [25]. PFS and OS are improved with multi-pathway therapy, such as simultaneous inhibition of VEGF- and mTOR-mediated pathways compared to single-pathway therapy.

The frequency of AE was significantly larger in the two study groups that included lenvatinib (either as monotherapy or combined with everolimus) compared to monotherapy with everolimus [31,35]. Grade 3- and 4-AE occurred in 79%, 71% and 50% of patients in the study groups with combined lenvatinib/everolimus, lenvatinib and everolimus, respectively. Two fatal AE were assessed to be study-related: One patient died from cerebral hemorrhage in the combined therapy group. Another patient died from myocardial infarction in the lenvatinib monotherapy group. The cause for these fatal events is unclear.

### 3.5. Multikinase Inhibitor-Related Hypertension as a Biomarker for Therapuetic Efficacy

The occurrence of sunitinib-induced hypertension has been shown to be associated with improved PFS and/or OS for patients with mRCC and has been proposed as an efficacy biomarker Rini et al. reported objective response rates of 54.8% vs. 8.7%, median PFS of 12.5 (95% CI: 10.9–13.7) vs. 2.5 (95% CI: 2.3–3.8) months and OS of 30.9 (95% CI: 27.9–33.7) vs. 7.2 (95% CI: 5.6–10.7) months for patients developing sunitinib-related hypertension vs. patients without hypertension [46]. Similar effects were observed by additional studies, which also found that the co-occurrence of more than one AE (for example hand-foot-syndrome, neutropenia or thrombocytopenia) is an even stronger predictor of prolonged survival than hypertension alone [47,48,49,50].

So far, no comparable data on lenvatinib and everolimus are available. However, given the comparable mode of action of lenvatinib and sunitinib, it will be an interesting question for future research and possible individualized therapy regimens using lenvatinb/everolimus in combination.

## 4. Strategies for Modifying the Effects of Hypertension Caused by Multikinase Inhibitors and mTOR Inhibitors

Severe arterial hypertension is causally linked to complications such as myocardial infarction, cerebral hemorrhage, and renal failure [2]. A timely and adequate treatment and/or prevention of arterial hypertension can reduce the frequency of these cardiovascular events.

Before initiating lenvatinib and/or everolimus therapy as well as during therapy, blood pressure must be controlled. Generally recommended are lifestyle modifications including dietary reduction of sodium and cholesterol intake or an increase of physical activity when possible. If the blood pressure is elevated, antihypertensive therapy must be initiated [9]. First-line antihypertensive therapeutics for mRCC patients include angiotensin-converting enzyme inhibitors (ACEi), angiotensin-receptor blockers (ARB), calcium channel inhibitors, and thiazide diuretics [2,51,52,53]. The most appropriate strategy of first-line antihypertensive pharmacotherapy depends on multiple variables such as co-morbidity (e.g., diabetes, renal disease) and patient-specific contraindications or other factors. If first-line antihypertensive pharmacotherapy, together with lifestyle modifications are not sufficient to control hypertension the second-line pharmacotherapy may be added including beta-adrenergic receptor blockers and/or aldosterone antagonists. Third line pharmacotherapy consists of the administration of long-lasting nitric oxide donors. Phosphodiesterase inhibitors can enhance the vasodilator effect of nitric oxide donors [2].

Grade 3-hypertension as an AE of lenvatinib therapy despite optimal antihypertensive treatment requires medical interventions with hold of the lenvatinib therapy in mRCC. When the hypertension resolves to grade 0, 1 or 2 (Figure 1), lenvatinib may be resumed with a reduced dose. In case of severe pharmacoresistant and/or life-threatening grade 3 hypertension, urgent intervention is mandated and the lenvatinib therapy is discontinued. FDA cautions against resuming the lenvatinib therapy [9]. It even recommends to first hold or discontinue lenvatinib and everolimus, when patients with mRCC receiving a combined treatment with lenvatinib and everolimus have hypertension [9].

For hypertension causally linked to everolimus alone, the FDA recommends to discontinue, interrupt, or use alternate day dosing [9]. The overall frequency of AE that require dose reduction or discontinuation of lenvatinib and everolimus therapy is 89% and 54% in patients treated with everolimus as monotherapy [9].

Evidence for the best antihypertensive strategy in mRCC treated with VEGF and mTOR inhibitors is very scarce. Recommendations for antihypertensive therapy during treatment of cancer tend to be based on general cardiology recommendations. However, arterial hypertension can be a surrogate marker of effective antitumor efficacy of VEGF therapy in mRCC, and can even be a predictor of improved clinical outcome in this patient population [2].

### Best Therapeutic Alternative

Cabozantinib is a promising multikinase inhibitor with an inhibitory effect on the VEGF pathway. However, the use of cabozantinib is also complicated by a high frequency of arterial hypertension [2]. A clinical comparison of cabozantinib and everolimus for treatment of mRCC demonstrated that the incidences and severities of therapy-related arterial hypertension were similar in the two treatment groups. Overall 37% of the patients developed arterial hypertension [2].

## 5. Discussion

Multikinase and mTOR inhibitors of signaling pathways of neoangiogenesis and tumor growth significantly and effectively improve PFS and OS in mRCC [54,55]. Unfortunately, these pharmaceutical agents are associated with numerous AE. A frequent and potentially severely comorbid and even fatal AE of combined treatment with lenvatinib and everolimus is arterial hypertension. Its pathogenesis during lenvatinib/everolimus treatment of mRCC is multifactorial.

At present, the strategy of antihypertensive treatment in terminally ill patients with mRCC complies with the general cardiovascular antihypertensive strategy and is supported by studies showing that co-treatment with any antihypertensiva do not diminish the anti-tumor activity of the tyrosine kinase inhibitor (TKI) sunitinib [56,57]. The question here is whether traditional cardiovascular predictors of negative effects of hypertension are relevant for the hypertensive mRCC subgroup of onco-cardiological patients, because the five-year survival rate for the mRCC group is extremely low compared to that of other groups of patients with hypertension.

Hypertension due to treatment of RCC with multikinase and mTOR inhibitors differs from other forms of hypertension in many ways. The various treatment-specific pathogenic factors may serve as potential selective targets of antihypertensive treatment in the future. Another future strategy may be the development of tools with high specificity and sensitivity for the risk prediction of severe hypertension to treatment with multikinase and mTOR inhibitors in RCC patients. Maybe patients prone to develop severe hypertension could benefit from antihypertensive therapy prior to the start of a treatment with multikinase and mTOR inhibitors. Anti-VEGF antibody induced hypertension apparently can be reduced by concomitant treatment with a nitric oxide donor, which can induce vasodilatation [2]. Future research is required to elucidate whether it is possible to design new multikinase/mTOR or other signaling pathway inhibitors that can improve PFS and OS in mRCC without causing arterial hypertension and other AE associated with increased morbidity and mortality. Whether the net effect of multikinase/mTOR inhibitor therapy versus manifest arterial hypertension favors the anticancer therapy depends mainly on the severity of hypertension and the effect of antihypertensive treatment and needs to be assessed clinically for each individual patient.

## 6. Conclusions and Outlook

Inhibitors of the VEGF and mTOR signaling pathways can reduce angiogenesis, tumor growth, and proliferation and thereby increase PFS as well as OS in mRCC. However, these targeted inhibitors of mRCC tumor growth are causally linked to cardiovascular complications—especially arterial hypertension, due to increased vascular tone, rarefaction and increased arterial stiffness. Severe hypertension can increase morbidity and risk of fatality, which mandates hold or even discontinuation of the anti-cancer therapy.

The pathogenesis of hypertension caused by inhibitors of the VEGF and mTOR signaling pathways may offer selective targets of antihypertensive treatment in the future. Another future perspective is development of new inhibitors of angiogenesis and tumor growth that are not associated with critical AE such as severe arterial hypertension. Further research is required to deal with this challenge.

## Figures and Tables

**Table 1:** Clinical and preclinical trials with lenvatinib and everolimus.

Study Title	Characteristics	Aim	Results
Targeting of tumor growth and angiogenesis underlies the enhanced antitumor activity of lenvatinib in combination with everolimus [26].	Preclinical study	To assess whether the combination of everolimus and lenvatinib showed greater antitumor effect than each of the single-agent treatments alone.	The combination therapy showed a greater antitumor effect due to an improved anti-angiogenic effect of lenvatinib and an anti-proliferative effect of everolimus.
Efficacy of everolimus in advanced renal cell carcinoma: a double-blind, randomized, placebo-controlled phase III trial NCT00410124 [30].	Randomized, double-blind, placebo-con- trolled, phase III trial	To assess improvement of progression-free survival (PFS) in metastatic renal cell carcinoma (mRCC) patients treated with everolimus after progression of disease during VEGF inhibition treatment.	Treatment with everolimus improved PFS in patients that had experienced progression of disease during vascular endothelial growth factor (VEGF) inhibition treatment.
Lenvatinib, everolimus and the combination in patients with metastatic renal cell carcinoma NCT01136733 [31].	Randomized, open-label, multicenter, phase II trial	To compare treatment of patients with the combination of everolimus and lenvatinib with single-agent treatment with lenvatinib and single-agent treatment with everolimus.	PFS was increased in both groups receiving lenvatinib compared to the group receiving everolimus. The benefit on PFS was more robust with the combination therapy. The incidence of hypertension was higher in the combination group (27%) and lenvatinib group (31%) compared to the everolimus group (8%).
A phase Ib clinical trial of the multi-targeted tyrosine kinase inhibitor lenvatinib (E7080) in combination with everolimus for treatment of metastatic renal cell carcinoma (RCC) NCT01136733 [36].	Open-label, phase Ib trial	To assess safety and antitumor activity of the combination therapy of everolimus and lenvatinib.	The clinical benefit of combination therapy with lenvatinib and everolimus showed to be favorable. Most of the adverse events (AEs) were due to class effects of VEGF and mammalian target of rapamycin (mTOR) inhibitors.
Cabozantinib versus everolimus in advanced renal cell carcinoma (METEOR): final results from a randomized, open label, phase 3 trial NCT01865747 [37].	Randomized, open-label, phase III trial	To compare the safety and efficacy of treatment with the mTOR inhibitor everolimus and the tyrosine-kinase inhibitor Cabozantinib.	Treatment with cabozantinib improved overall survival (OS) significantly compared to everolimus. Median survival was 21.4 months in patients treated with cabozantinib and 16.5 months in patients treated with everolimus. The incidence of grade 3 or 4 AE was higher in the group treated with cabozantinib (71%) than the group treated with everolimus (60%). The most common grade 3 or 4 AE in the cabozantinib group was hypertension (15% vs. 4% in the everolimus group).

## Abbreviations

AE	adverse event
ccRCC	clear cell renal cell carcinoma
eNOS	endothelial nitric oxide synthase
FDA	food and drug administration
HUVEC	human umbilical vein endothelial cells
IGF-1	insulin-like growth factor type 1
MTD	maximum tolerated dose
mRCC	metastatic renal cell carcinoma
mTOR	mammalian target of rapamycin
mTORC1	mammalian target of rapamycin complex 1
mTORC2	mammalian target of rapamycin complex 2
NO	nitric oxide
NOS	nitric oxide synthase
OS	overall survival
PFS	progression free survival
pVHL	von Hippel-Lindau protein
RCC	renal cell carcinoma
RCT	randomized controlled trial
RTK	receptor tyrosine kinase
TNM	cancer staging classification based on tumor/nodes/metastases
VEGF	vascular endothelial growth factor
VEGFR1	vascular endothelial growth factor receptor 1
VEGFR2	vascular endothelial growth factor receptor 2
VEGFR3	vascular endothelial growth factor receptor 3
VHL	von Hippel-Lindau
